# Robotic-Assisted Retromuscular Umbilical Prosthetic Hernia Repair (r-TARUP) With Hugo-RAS System: Case Series and Technique

**DOI:** 10.1097/SLE.0000000000001409

**Published:** 2025-09-24

**Authors:** Richard Sassun, Pietro Achilli, Lorenzo Morini, Francesco Brucchi, Vincenzo Nicastro, Giuseppe di Donna, Riccardo Magarini, Bruno Domenico Alampi, Giovanni Ferrari

**Affiliations:** * University of Milan; † Department of Mininvasive Oncologic Surgery, ASST Grande Ospedale Metropolitano Niguarda, Milan, Italy

**Keywords:** robotic, hugo, hernia, umbilical, retromuscular

## Abstract

**Background:**

Minimally invasive approaches have transformed ventral hernia management, with robotic platforms enhancing challenging techniques like the robotic Transabdominal Retromuscular Umbilical Prosthesis (r-TARUP). While traditionally performed using the da Vinci system, the introduction of the Medtronic Hugo RAS system offers a valuable alternative. We present our first case series with standardized surgical technique for r-TARUP using the Hugo RAS system, detailing operative setup, technical considerations, and initial outcomes.

**Methods:**

Between September 2024 and February 2025, we performed 30 r-TARUP procedures using the Hugo RAS system. Preoperative evaluation included imaging and risk factor optimization. The technique involved a lateral retromuscular approach, ipsilateral posterior rectus sheath (PRS) closure, and mesh placement. Patients were discharged the following day, with follow-ups assessing complications and recurrences.

**Results:**

The mean hernia dimensions were 3.1±1.0 cm in width and 2.6±0.8 cm in length. Rectus diastasis repair was performed in 57% of cases. Mean operating and docking times were 190.6±61.6 and 15±5.3 minutes, respectively. Two cases required conversion to laparoscopic surgery during peritoneal closure. No complications or recurrences were observed after a mean follow-up of 3.6 (1.7 to 5.3) months.

**Conclusions:**

Despite the short follow-up, our experience demonstrates the feasibility and safety of r-TARUP with the Hugo RAS system. Comparable outcomes to other robotic platforms suggest that the Hugo RAS system is a viable alternative for ventral hernia repair, offering technical flexibility and promising short-term results.

The management of ventral hernias has undergone significant evolution over the last few decades, with a shift towards minimally invasive approaches aimed at reducing morbidity and improving outcomes, as evidenced by multiple studies.^1–3^ Traditional laparoscopic ventral hernia repair often entails the placement of an intraperitoneal mesh, which, despite its initial efficacy, is associated with long-term complications such as adhesion formation and increased complexity during subsequent abdominal surgeries.^4–6^ Furthermore, fixation of intraperitoneal meshes using tacks or transabdominal sutures has been linked to both short-term and long-term postoperative pain.^7^

These challenges have spurred the development of alternative approaches, such as the laparoscopic retromuscular mesh repair, which has demonstrated superior outcomes compared with intraperitoneal mesh placement.^8,9^ By accessing the retromuscular plane through a lateral approach, the hernia is reduced while avoiding complications related to intraperitoneal mesh. However, this technique was deemed technically demanding, particularly for steps such as defect closure and suturing of the ipsilateral posterior rectus sheath.

The advent of robotic platforms has significantly facilitated these challenging steps. Many authors further refined this approach in 2018 with the robotic Transabdominal Retromuscular Umbilical Prosthesis (r-TARUP), which demonstrated reduced operative times compared with the laparoscopic technique, low conversion rates, and low complications and recurrence rates.^10^ This technique was carried out in other scenario such as rectus diastasis and parastomal hernias, revealing better outcomes than intraperitoneal meshes.^11–13^

Robotic platforms have gained widespread acceptance for these procedures, historically dominated by Intuitive Surgical’s da Vinci systems. However, the introduction of new robotic platforms, such as the Medtronic Hugo RAS system, has broadened the technological landscape. The Hugo RAS system, with its modular design and separate arm carts, provides enhanced adaptability for surgical procedures and operating room configurations.^14,15^

Given the increasing use of the Hugo RAS system, we present our first case series with a standardized surgical technique for r-TARUP performed using the Medtronic Hugo RAS system. We aim to detail the technical considerations, including operating room setup, trocar placement, and arm positioning, as well as to evaluate the feasibility and safety of this novel robotic approach.

## MATERIAL AND METHODS

### Study Design and Patient Selection

**FIGURE 1:**
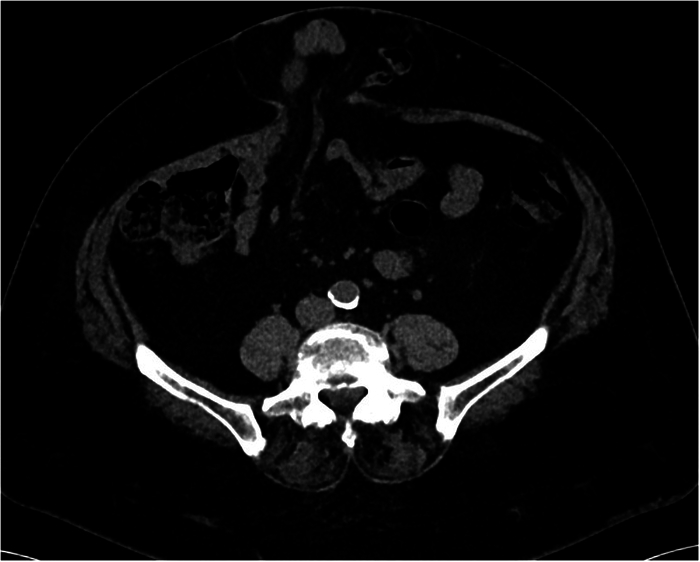
Computed tomography imaging of an umbilical hernia.

This is a prospective, single-center, nonsponsored, observational cohort study on the experience with r-TARUP using the Hugo RAS system in a consecutive initial series of 30 operations. Consecutive patients scheduled for elective treatment of an umbilical hernia, both primary and incisional, with a minimally invasive technique, were eligible for a robotic-assisted approach. Preoperative evaluation includes detailed medical and surgical history, physical examination, and preoperative cross-sectional imaging. Smoking cessation, physical exercise, and weight loss are recommended to all patients. Despite this, all these factors, as well as previous abdominal surgery, are not considered exclusion criteria for a r-TARUP. Patients were excluded if the size of the defect was higher than 5 cm in width due to the limited experience with this new robotic platform (Fig. 1). All the surgical interventions were performed by 2 surgeons who had completed the official technical training on Hugo RAS technology. This study was approved by the ethics committees of ASST Grande Ospedale Metropolitano Niguarda (protocol number 73-25012023).

### Instrumentation

The robotic instruments used include the Large Needle Driver, Monopolar Curved Scissors, and Fenestrated Bipolar Forceps. Additional accessories include nonabsorbable polyvinylidene fluoride (PVDF) mesh (DynaMesh-CICAT), barbed resorbable sutures (0 and 3/0), and a glue for mesh fixation.

### Patient Positioning and Setup

**FIGURE 2:**
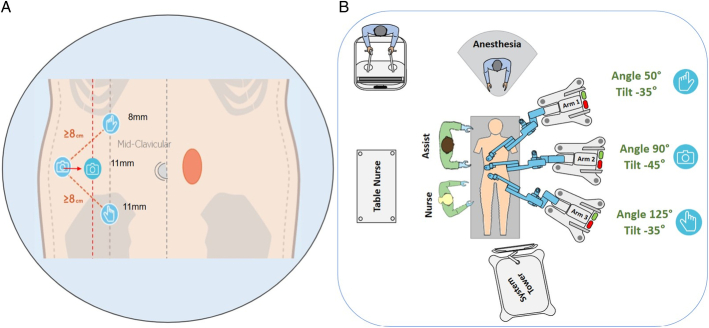
Trocar layout and OR setup showing docking and tilt angles with Hugo RAS platform.

Patients are placed in the supine position with both arms tucked alongside the body to maximize workspace for the robotic platform. A transversus abdominis plane block is usually performed by an anesthesiologist. The 3 robotic arm carts are positioned on the patient’s left side, while trocars are placed on the right side. Pneumoperitoneum is established at 12 mm Hg using a Veress needle. Two 11 mm robotic trocars and one 8 mm trocar are inserted along the right anterior axillary line (Fig. 2). The subcostal trocar is positioned at the level of the anterior axillary line, with 2 additional trocars spaced at least 8 cm apart caudally. The docking of the Hugo robotic arms is completed, ensuring the endoscope occupies the central trocar. A 0-degree camera is utilized for the whole procedure. The camera arm is set with a 45 degree angle having a 90 degree angle with the surgical table, while the remaining operative arms are set with a 50 degree angle having a 35 and 125 degree angle with the surgical table, for the left and right hand, respectively (Fig. 2). This differs from the Medtronic’s specialists’ suggestions on the tilt and table angles.

### Surgical Procedure

**FIGURE 3:**
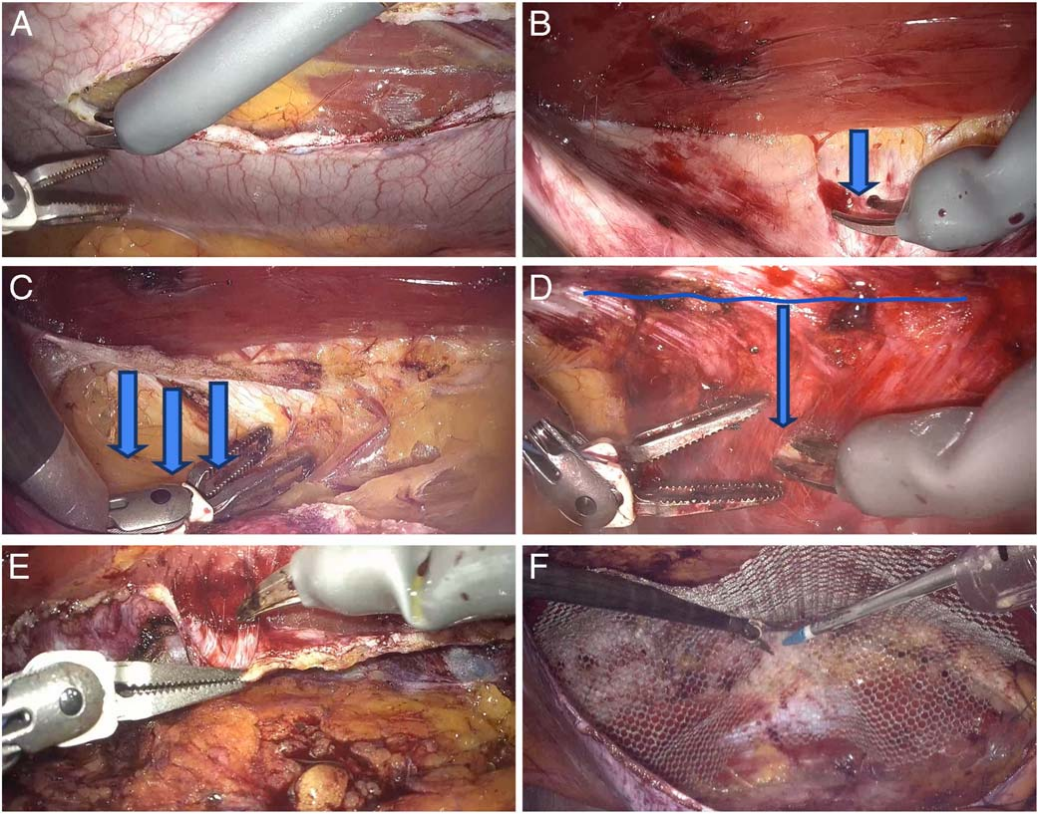
Surgical steps for r-TARUP. (A) Identification of the lateral margin of the rectus muscle and initiation of dissection cranially and caudally. (B) Incision of the posterior rectus fascia about 1 cm from the medial margin of the rectus muscle, carefully sparing the linea alba. (C) After dissecting and moving the preperitoneal fat downward, the contralateral posterior fascia is identified. (D) Incision of the posterior fascia of the contralateral rectus muscle, maintaining a few centimeters distance from the linea alba. (E) Prepare the contralateral retromuscular space. (F) Fix the mesh starting from the midline and then securing the sides.

The hernia sac and contents are reduced carefully while preserving the peritoneum. The ipsilateral posterior rectus sheath (PRS) is opened 5 cm lateral to the hernia defect (Fig. 3A). The medial border of the rectus muscle is exposed, followed by the longitudinal incision of the PRS near its junction with the linea alba (Fig. 3B). Dissection proceeds in a lateral-to-medial fashion, ensuring entry into the preperitoneal space. Cranial and caudal planes are developed, identifying the contralateral posterior fascia by moving the preperitoneal fat downward (Fig. 3C). Transabdominal spinal needles guide PRS incision orientation to prevent deviation.

The contralateral PRS is incised 5 mm lateral to the linea alba to perform retromuscular dissection, identifying neurovascular bundles and the semilunar line (Fig. 3D). The hernia defect is closed with a running barbed suture after lowering the pneumoperitoneum to 8 mm Hg. In cases of diastasis recti, horizontal mattress sutures are used to reduce postoperative midline ridging.

The contralateral retromuscular space is prepared, ensuring adequate overlap for mesh placement (Fig. 3E). A PVDF mesh is introduced into the retromuscular space through the right-arm 11 mm trocar. The mesh is oriented using the large needle driver and the bipolar forceps. Fixation with glue is performed by the assistant with cyanoacrylate glue and through the right arm trocar (Fig. 3F). The ipsilateral PRS and peritoneum are closed using a 3/0 barbed resorbable suture. The abdominal fascia where the 11 mm robotic trocars are placed is closed using a 3/0 Vicryl suture.

### Postoperative Management

The nasogastric tube and the Foley catheter are removed in the operating room. We asked patients to wear abdominal binder for the first 6 weeks after surgery, which reduces pain, recurrences, and seromas rate. A regular diet is given the same day, and the patient is discharged the next day with a scheduled postoperative ambulatory visit. Cross-sectional imaging is obtained in case of suspected recurrent hernia or complications.

### Outcomes

The outcomes studies were skin-to-skin operative time, conversion to laparoscopic surgery, seromas, hematomas, and recurrence rates, assessed with an objective examination and, if needed, an ultrasound or computed tomography.

### Statistical Analysis

Categorical variables were reported as frequencies (percent), while continuous variables were reported as mean±SD or median (interquartile range) according to their distribution. Missing values were excluded from the descriptive analyses. All analyses were conducted using the Statistical Package for Social Sciences (SPSS version 28).

## RESULTS

We performed 30 r-TARUP using the abovementioned surgical technique with the Medtronic Hugo RAS system in our institution between September 2024 and February 2025. Patient characteristics are illustrated in Table 1 and compared with literature in Table 2.

**TABLE 1:** Patients’ Characteristics and Outcomes

Characteristic	n=30, n (%)
Age, y, mean (SD)	58 (14.1)
Male	18 (60)
BMI, kg/m^2^, mean (SD)	28.4 (5.6)
Hernia width, cm, mean (SD)	3.1 (1.1)
Hernia length, cm, mean (SD)	2.6 (1.0)
Rectus diastasis repair	17 (57)
Skin-to-skin operative time, min, mean (SD)	190.6 (61.6)
Docking time, minutes, mean (SD)	15 (5.3)
Conversion to laparoscopic surgery	2 (7)
Length of stay, d, mean (SD)	1.1 (0.3)
Seromas	0
Hematomas	0
Recurrence	0
Follow up, mo, median (IQR)	3.6 (1.7-5.3)

**TABLE 2:** Literature Review of r-TARUP Outcomes

Characteristic	Sassun (%)	Ferraro (%)	Muysoms (%)	Cuccurullo (%)	Baur (%)	Garza (%)	Jaro	Vierstraete (%)
N	30	39	41	45	30	101	62	80
Operative time	191 (62)	137 (32)	114 (19)	188 (111)	109 (32)		146 (121)	73 (19)
Conversion to laparoscopy	7	0		0				4
Length of stay	1.1 (0.3)	1.7 (0.9)		4.4 (3.8)	2.7 (1.7)			
Infection			2	2	3			0
Seroma	0	5	5		23	2		5
Hematoma	0	0	2		10	3		0
30 d readmission	0	0	0					
Recurrence	0	0	0	9	0	3		3
Follow up, mo	3.6	21	1	12	1.5			60

The mean age was 58±14.1 years, 18 (60%) patients were male, and the mean body mass index (BMI) was 28.4±5.6 kg/m^2^. The mean hernia width was 3.1±1.1 cm, while the mean hernia length was 2.6±1.0 cm. Seventeen (57%) patients showed a rectus diastasis, which was repaired with an inward plication using a horizontal mattress suture. Mean skin-to-skin operating time was 190.6±61.6 minutes, while the mean docking time was 15±5.3 minutes. Only 2 (7%) cases were converted to laparoscopic surgery during the peritoneal closure phase. The mean length of stay was 1.1±0.3 days. No seromas, hematomas, surgical-site infections (SSIs), or recurrences were reported after a median follow-up of 3.6 (1.7 to 5.3) months.

## DISCUSSION

Our study highlights the feasibility and safety of robotic-assisted transabdominal retromuscular umbilical prosthetic hernia repair (r-TARUP) using the Medtronic Hugo RAS system. The findings demonstrate comparable outcomes to previously reported results with other robotic platforms, indicating that this newer robotic system is a viable alternative for ventral hernia repair.

One of the key advantages of the Hugo RAS system is its modular design, which allows for greater adaptability in operating room setups. Unlike other robotic platforms with fixed robotic arms, the Hugo RAS system consists of independent robotic arms that can be positioned more flexibly. This setup can be particularly beneficial in institutions with space constraints or those aiming to optimize resource allocation.

Our operative times, with a mean skin-to-skin duration of 190 minutes, align with previous reports on r-TARUP procedures performed with other robotic platforms. A study by Ferraro et al^16^ revealed a mean operative time of 154 minutes during transabdominal retromuscular midline hernias (TARM) repair with the DaVinci Xi, which is comparable to ours. Their seroma, hematoma, and recurrence rates are comparable to our results, supporting the reproducibility of this technique in experienced hands.

Only Muysoms et al^10^ reported a mean skin-to-skin duration of 81 minutes with the DaVinci Xi system, which was reduced to 61 minutes after some experience. Their faster operative time could be explained by several factors. First, the omission of mesh fixation with glue, which takes about 10 minutes. Second, rectus diastasis was not repaired in their cohort, while 56% of our cohort did. Third, a faster docking time, as our mean docking time is longer than the one reported by Muysoms, although this is expected with the adoption of new technology and is likely to decrease with further experience. Lastly, a larger sample size which allows for more surgical practice and a decreased operative time.

An important consideration in the adoption of robotic platforms is the associated cost and accessibility. The DaVinci system has long dominated the robotic surgery market but is often associated with high acquisition and maintenance costs, which may limit its widespread adoption, particularly in resource-constrained settings. In contrast, the Medtronic Hugo RAS system was developed with a focus on affordability and market competition. Its modular design and independent arm carts not only offer greater flexibility in operating room configuration but also present a potentially lower cost of entry for institutions seeking to establish or expand robotic surgery programs.

The observed benefits of the r-TARUP technique over traditional laparoscopic repairs include faster operative time, reduced complications, enhanced visualization and improved ergonomics, which facilitate meticulous peritoneal closure and secure mesh placement.^17,18^ These are expected improvements, as other complex surgeries demonstrate benefit with a robotic approach.^1,19^ The retromuscular positioning of the mesh eliminates the need for transabdominal fixation, potentially reducing postoperative pain and long-term complications such as adhesions and bowel erosion. In addition, the ability to perform concurrent rectus diastasis repair in 57% of cases underscores the versatility of this approach in addressing midline defects.

This study reports the first case series with the Hugo RAS system for r-TARUP. Despite the promising results, our study has limitations. The small sample size and short follow-up duration limit long-term outcome assessments, including recurrence rates beyond the 3-month period. Moreover, while our initial findings demonstrate the feasibility of using the Hugo RAS system for r-TARUP, future studies comparing clinical outcomes, cost-effectiveness, and long-term durability between different robotic platforms will be essential to establish its definitive role in hernia repair.

In conclusion, we described our surgical technique for r-TARUP with the Medtronic Hugo RAS system. The results are comparable to the ones previously reported for r-TARUP, suggesting that this procedure is safe and replicable. A longer follow-up is needed to assess recurrences for this series.

## References

[1] Deerenberg EB Henriksen NA Antoniou GA . Updated guideline for closure of abdominal wall incisions from the European and American Hernia Societies. Br J Surg. 2022;109:1239–1250. 36026550 10.1093/bjs/znac302 PMC10364727

[2] Mason RJ Moazzez A Sohn HJ . Laparoscopic versus open anterior abdominal wall hernia repair: 30-day morbidity and mortality using the ACS-NSQIP database. Ann Surg. 2011;254:641–652. 21881493 10.1097/SLA.0b013e31823009e6

[3] Martins MR Santos-Sousa H do Vale MA . Comparison between the open and the laparoscopic approach in the primary ventral hernia repair: a systematic review and meta-analysis. Langenbecks Arch Surg. 2024;409:52. 38307999 10.1007/s00423-024-03241-y PMC10837225

[4] Jenkins ED Yom V Melman L . Prospective evaluation of adhesion characteristics to intraperitoneal mesh and adhesiolysis-related complications during laparoscopic re-exploration after prior ventral hernia repair. Surg Endosc. 2010;24:3002–3007. 20445995 10.1007/s00464-010-1076-0

[5] Fortelny RH Petter-Puchner AH Glaser KS . Adverse effects of polyvinylidene fluoride-coated polypropylene mesh used for laparoscopic intraperitoneal onlay repair of incisional hernia. Br J Surg. 2010;97:1140–1145. 20632284 10.1002/bjs.7082

[6] Sharma A Chowbey P Kantharia NS . Previously implanted intra-peritoneal mesh increases morbidity during re-laparoscopy: a retrospective, case-matched cohort study. Hernia. 2018;22:343–351. 29151228 10.1007/s10029-017-1686-8

[7] Calpin GG Davey MG Whooley J . Evaluating mesh fixation techniques for ventral hernia repair: a systematic review and network meta-analysis of randomised control trials. Am J Surg. 2024;228:62–69. 37714741 10.1016/j.amjsurg.2023.09.015

[8] Kudsi OY Kaoukabani G Bou-Ayash N . Clinical outcomes and costs of retromuscular and intraperitoneal onlay mesh techniques in robotic incisional hernia repair. Surg Endosc. 2024;38:2850–2856. 38568440 10.1007/s00464-024-10776-0

[9] Jensen KK Helgstrand F Henriksen NA . Short-term outcomes after laparoscopic IPOM versus robot-assisted retromuscular repair of small to medium ventral hernias: a nationwide database study. Ann Surg. 2024;279:154–159. 37212128 10.1097/SLA.0000000000005915

[10] Muysoms F Van Cleven S Pletinckx P . Robotic transabdominal retromuscular umbilical prosthetic hernia repair (TARUP): observational study on the operative time during the learning curve. Hernia. 2018;22:1101–1111. 30244344 10.1007/s10029-018-1825-x

[11] Cuccurullo D Guerriero L Mazzoni G . Robotic transabdominal retromuscular rectus diastasis (r-TARRD) repair: a new approach. Hernia. 2022;26:1501–1509. 34982294 10.1007/s10029-021-02547-w

[12] Maciel V Mata W Arevalo G . Robotic retro-rectus repair of parastomal hernias. J Robot Surg. 2019;13:483–489. 30251135 10.1007/s11701-018-0874-6

[13] Violante T Ferrari D Gomaa IA . Robotic parastomal hernia repair in Ileal-conduit patients: short-term results in a single-center cohort study. Hernia. 2024;28:2245–2253. 39240470 10.1007/s10029-024-03153-2

[14] Rottoli M Violante T Calini G . A multi-docking strategy for robotic LAR and deep pelvic surgery with the Hugo RAS system: experience from a tertiary referral center. Int J Colorectal Dis. 2024;39:154. 39349880 10.1007/s00384-024-04728-2 PMC11442597

[15] Belyaev O Fahlbusch T Slobodkin I . Use of HugoTM RAS in general surgery: the first 70 cases at a german centre and a systematic review of the literature. J Clin Med. 2024;13:3678. 38999244 10.3390/jcm13133678 PMC11242108

[16] Ferraro L Formisano G Salaj A . Robotic trans-abdominal retromuscular hernia repair for medium-sized midline hernias: midterm outcomes and surgical site occurrence (SSO) analysis in 120 patients. J Robot Surg. 2025;19:1–9. 10.1007/s11701-024-02184-2 39680358

[17] Masurkar AA . Laparoscopic trans-abdominal retromuscular (TARM) repair for ventral hernia: a novel, low-cost technique for sublay and posterior component separation. World J Surg. 2020;44:1081–1085. 31773221 10.1007/s00268-019-05298-z

[18] Rege SA Churiwala JJ A Kaderi AS . Comparison of efficacy and safety of the enhanced-view totally extraperitoneal (eTEP) and transabdominal (TARM) minimal access techniques for retromuscular placement of prosthesis in the treatment of irreducible midline ventral hernia. J Minim Access Surg. 2021;17:519. 33885011 10.4103/jmas.JMAS_145_20 PMC8486046

[19] Sassun R Sileo A Ng JC . Diverticular disease complicated by colovesical and colovaginal fistulas: not so complex robotically. Surgical Endoscopy. 2025;39:3941–3946. 40355739 10.1007/s00464-025-11754-w

